# On Neural Networks Fitting, Compression, and Generalization Behavior via Information-Bottleneck-like Approaches

**DOI:** 10.3390/e25071063

**Published:** 2023-07-14

**Authors:** Zhaoyan Lyu, Gholamali Aminian, Miguel R. D. Rodrigues

**Affiliations:** 1 Department of Electronic and Electrical Engineering, University College London, Gower St., London WC1E 6BT, UK; m.rodrigues@ucl.ac.uk; 2 The Alan Turing Institute, British Library, 96 Euston Rd., London NW1 2DB, UK; gaminian@turing.ac.uk

**Keywords:** deep learning, information theory, information bottleneck, generalization, fitting, compression

## Abstract

It is well-known that a neural network learning process—along with its connections to fitting, compression, and generalization—is not yet well understood. In this paper, we propose a novel approach to capturing such neural network dynamics using information-bottleneck-type techniques, involving the replacement of mutual information measures (which are notoriously difficult to estimate in high-dimensional spaces) by other more tractable ones, including (1) the minimum mean-squared error associated with the reconstruction of the network input data from some intermediate network representation and (2) the cross-entropy associated with a certain class label given some network representation. We then conducted an empirical study in order to ascertain how different network models, network learning algorithms, and datasets may affect the learning dynamics. Our experiments show that our proposed approach appears to be more reliable in comparison with classical information bottleneck ones in capturing network dynamics during both the training and testing phases. Our experiments also reveal that the fitting and compression phases exist regardless of the choice of activation function. Additionally, our findings suggest that model architectures, training algorithms, and datasets that lead to better generalization tend to exhibit more pronounced fitting and compression phases.

## 1. Introduction

Deep learning models have gained enormous attention thanks to their impressive performance compared with traditional learning models in a variety of areas, such as computer vision, speech processing, natural language processing, and many more [1,2]. However, despite their stunning performance, we still do not fully understand how deep neural networks work [3].

A number of recent approaches have been proposed to study the generalization/ optimization properties of over-parameterized models, such as deep neural networks [4,5]. However, these approaches do not fully capture certain neural network representation properties, including how these evolve during the neural network training procedure. Such an understanding of the role of different components of the model and their impact on the learning process can be essential for selecting or designing better neural network models and associated learning algorithms.

Another popular approach to studying the generalization/optimization dynamics of deep neural networks has been the information bottleneck (IB). This approach, which is based on the information bottleneck theory [6,7], employs the mutual information (MI) between the data and their neural network representation, as well as MI between labels and the neural network representation to capture neural network behavior. In particular, in classification problems, it is typical to model the relationship between the data label *Y*, the data themselves *X*, and some neural network intermediate data representation *Z* via a Markov chain Y→X→Z, where *Y*, *X*, and *Z* represent random variables/vectors associated with these different objects. Then, the IB principle is described via two MIs: (1) I(Z;X) to measure the amount of information contained in the data representation about the input data, and (2) I(Z;Y) to measure the information in the data representation that could contribute to the prediction of ground-truth labels. One can capture how the value of I(Z;X) and I(Z;Y) evolve as a function of the number of training epochs for a neural network by plotting pairs of these mutual information values on a two-dimensional plane [8]. The plane defined by these MI terms is called the information plane (IP), and the trace of the MI value versus training epoch is called the information plane dynamic (IP-dynamic).

This approach has led to the identification of some trends associated with the optimization of neural networks. In particular, by observing the IP-dynamic of the networks trained on a synthetic dataset and the MNIST dataset, ref. [8] found that, in early epochs, both I(Z;X) and I(Z;Y) increase; and, in later epochs, I(Z;Y) will keep increasing while I(Z;X) decreases. This led to the conjecture that the training of a neural network contains two different phases: (1) a **fitting phase,** where the network representation *Z* fits the input data *X* as much as possible, and (2) a subsequent **compression phase** in which the network compresses the useless information in the representation *Z* about the labels *Y*.

However, the IB approach requires estimating I(Z;X) and I(Z;Y), which is notoriously difficult to accomplish because the inputs and representations typically lie in very high-dimensional spaces. For example, non-parametric mutual information estimators—such as [9,10]—suffer from either high bias or high variance, especially in high-dimensional settings [10]. This will directly affect any conclusions extracted from the IP-dynamics because high bias prevents recognizing the existence of fitting or compression phases, whereas high variance leads to inconsistent results across different numerical experiments. Indeed, with different mutual information estimators, researchers drew diverse or opposite conclusions about trends in IP-dynamics [8,11,12,13,14,15,16,17,18,19,20,21,22,23,24,25]. For instance, Saxe et al. [24] argued that the reported phenomena of fitting and compression in Shwartz et al.’s study [8] are highly dependent on the simple binning MI estimator setup adopted.

Therefore, the trends that one often extracts from an IB analysis may not always hold.

### 1.1. Paper Contributions

This paper attempts to resolve these issues by introducing a different approach to studying the dynamics of neural networks. Our main contributions are as follows:

1 First, we propose to use more tractable measures to capture the relationship between an intermediate network data representation and the original data or the intermediate network representation and the data label. In particular, we used the minimum mean-squared error between the intermediate data representation and the original data to try to capture fitting and compression phenomena occurring in a neural network; we also used the well-known cross-entropy between the intermediate data representation and the data label to capture performance.
2 Second, by building upon the variational representations of these quantities, we also propose to estimate such measures using neural networks. In particular, our experimental results demonstrate that such an approach leads to consistent estimates of the measures using different estimator neural network architectures and initializations.
3 Finally, using our proposed approach, we conducted an empirical study to reveal the influence of various factors on neural network learning processing, including compression, fitting, and generalization phenomena. Specifically, we considered the impact of (1) the machine learning model, (2) the learning algorithm (optimizer and regularization techniques), and (3) the data.

The main findings deriving from our empirical study—along with the literature that explored similar network architecture, training algorithm, or data setups—are summarized in Table 1. In particular, we highlight that our study suggests that (1) a neural network generalization performance improves with the magnitude of the network’s fitting and compression phase; (2) a network tends to undergo a fitting phase followed by a compression phase, regardless of the activation function; and (3) the specific behavior of the fitting/compression phases depends on a number of factors, including the network architecture, the learning algorithm, and the nature of the data.

### 1.2. Scope of Study

Finally, we note that the information bottleneck technique has been used as a tool to cast insight into other machine learning paradigms, including semi-supervised learning [30] and unsupervised learning [31,32,33]. However, we focused exclusively on supervised learning settings—with an emphasis on neural networks—in order to contribute to a deeper understanding of deep learning techniques.

### 1.3. Paper Organization

This paper is organized as follows: Section 2 offers an overview of the literature that relates to our work. Section 3 proposes our approach to studying the compression, fitting, and generalization dynamics of neural networks, whereas Section 4 discusses practical implementation details associated with our proposed approach. Section 5 leverages our approach to conducting an empirical study of the impact of various factors on the compression, fitting, and generalization behavior of a neural network, including the underlying architecture, learning algorithm, and nature of the data. Finally, we summarize the paper, discuss its limitations, and propose future directions in Section 6.

### 1.4. Paper Notation

We adopt the following convention for random variables and their distributions throughout the paper. A random variable (or vector) is denoted by an upper-case letter (e.g., *Z*), and its space of possible values is denoted with the corresponding calligraphic letter (e.g., Z). The probability distribution of the random variable *Z* is denoted by $P_{Z}$. The joint distribution of a pair of random variables $\left(Z_{1},Z_{2}\right)$ is denoted by $P_{Z_{1},Z_{2}}$. H(Z) represents the entropy (or differential entropy) of random variable *Z*, $H(Z_{1}|Z_{2})$ represents the entropy (or differential entropy) of random variable $Z_{1}$ given random variable $Z_{2}$, and $I(Z_{1};Z_{2})$ represents the mutual information between random variables $Z_{1}$ and $Z_{2}$. We denote the set of integers from 1 to *n* by [n]≜{1,⋯,n}.

## 2. Related Work

There are various lines of research that connect to our work.

**Information bottleneck (IB) and information plane (IP) dynamics:** Many works have adopted the IB and the IP to study the optimization dynamics of neural networks. Refs. [8,18,19,26,28] concluded that there is a different fitting and compression phase during the training of a deep neural network, while [24,34] claim that neural networks with saturating activation functions exhibit a fitting phase but do not exhibit a compression phase. Ref. [11] conveyed that the network may occasionally compress only for some random initializations. On the other hand, ref. [11] found that weight decay regularization will increase the magnitude of the compression, while [14] did not observe compression unless weight decay is applied. Finally, overfitting was observed from the IP associated with hidden layers in [8,23,34].

While these works mentioned above explore various aspects of deep learning techniques, such as how network behaviors are affected by varying training dataset sizes and regularization techniques, their conclusions may not always be reliable due to the fact that MI estimation can be inaccurate and unstable in high-dimensional settings, as argued in [12].

**IB and IP based on other information measures:** Many works have also adopted IBs/IPs based on other information measures to study the dynamics of neural networks. Motivated by source coding, ref. [35] proposes to replace the I(Z;X) with the entropy of the representation *Z*. The authors in [36] introduced a generalized IB based on *f*-divergence. The authors also proposed an estimation bottleneck based on $\chi^{2}$-information, but this quantity is difficult to estimate in practice, preventing its applicability in various problems. The paper [37] proposed an information bottleneck approach based on MMSE and Fisher information to develop robust neural networks. However, the authors utilized MMSE to substitute mutual information between the representation and ground truth label, whereas we employed it to evaluate the association between representation and data. Inspired by [38], ref. [39] introduced a new IB—called the V-information bottleneck—that articulates the amount of useful information a representation embodies about a target usable by a classifier drawn from a family of classifiers V. Recently, refs. [40,41] have used sliced mutual information to study fitting in neural networks. However, their work mainly focused on the fitting phase and did not explore the role of compression and its relationship with generalization.

**Mutual information estimation:** Relying on mutual information to study the dynamics of neural networks leads to various challenges. The first challenge relates to the fact that the MI between two quantities that lie in continuous space and are linked by a functional relationship, such as the input and the output of a neural network, is theoretically infinite [42]. This limits its use since a neural network representation is typically a deterministic function of the neural network input [8,11,21,24]. Many works have circumvented this issue by adding additional noise to the random variables. For instance, kernel density estimation (KDE) [43,44] was used by [11,13,24,45], and the *k*-nearest-neighbor based Kraskov estimator [46] was used in [18,24,47]. Other works using variational mutual information estimators address the challenge by adding noise to the neural network representations [14,19]. However, adding noise to the representations of a neural network is not a widespread practice in most deep learning implementations. An alternative measure of dependence between two variables is sliced mutual information, which was proposed by [48]. This method involves random projections and the averaging of mutual information across pairs of projected scalar variables. Our approach differs from this method as we directly processed the random variables in high-dimensional space.

The second challenge relates to the fact that many mutual information estimators exhibit high bias and/or high variance in a high-dimensional setting. For example, simple binning methods [8,49] are known to lead to mutual information estimates that vary greatly depending on the bin size choice. Further, variational mutual information estimators, such as MINE [9], are also known to produce mutual information estimates that suffer from high bias or high variance [10,50].

Our work departs from existing work because we propose to study the evolution of two more stable measures during a neural network optimization process: (1) the minimum mean-squared error associated with the estimation of the original data given some intermediate network representation and (2) the cross-entropy associated with the original data label given an intermediate data representation. This offers a more reliable lens for studying compression, fitting, and generalization phenomena occurring in neural networks.

## 3. Proposed Framework

We now introduce our approach to studying the compression, fitting, and generalization dynamics of neural networks. We focused exclusively on classification problems characterized by a pair of random variables {(X,Y)|X∈X,Y∈Y}, where *X* is the input data and *Y* is the ground-truth label, that follow a distribution $P_{X,Y}$. We delivered an estimate of the ground-truth label $\hat{Y}\in Y$ given the data X∈X using an *L*-layer neural network as follows:

(1) $$\hat{Y}=f_{\theta }\left(X\right)=f_{\theta_{L}}^{\left(L\right)}\left(f_{\theta_{L-1}}^{\left(L-1\right)}\left(\cdots f_{\theta_{1}}^{\left(1\right)}\left(X\right)\right)\right)$$

where $f_{\theta_{l}}^{\left(l\right)}\left(\cdot \right)$ models the operation of the *l*-th (l∈[L]) network layer, where $\theta_{l}$ represents the parameters of this layer (the weights and biases). The network parameters were optimized using standard procedures given a (training) dataset containing various (training) samples.

The optimized network can then be used to make new output predictions $\hat{Y}$ given new input data *X*.

The network optimization procedure involves the application of iterative learning algorithms such as stochastic gradient descent. Therefore, at a certain epoch *i* associated with the learning algorithm, we can model the flow of information in the neural network via a Markov chain as follows:

(2) $$\begin{array}{c} Y\to X\to Z_{1}^{\left(i\right)}\to Z_{2}^{\left(i\right)}\to \cdots \to Z_{L}^{\left(i\right)}\to \hat{Y} \end{array}$$

where the random variable $Z_{l}^{\left(i\right)}=f_{\theta_{l}}^{\left(l\right)}\left(Z_{l-1}^{\left(i\right)}\right)\in R^{n_{l}}$ represents the network representation at layer *l* at epoch *i* in the $n_{l}$-dimension (with a convention that $Z_{0}^{\left(i\right)}=X$). Our goal was to examine how certain quantities—capturing the compression, fitting, and generalization behavior—associated with the network optimization process evolve as a function of the number of algorithm training epochs.

**Z-X measure:** Our first quantity describes the difficulty in recovering the original data *X* from some intermediate network representation $Z_{l}^{\left(i\right)}$ as follows:

(3) $$\begin{array}{cc} m_{Z_{l}^{\left(i\right)};X} & =\underset{f_{x}\in C\left(R^{n_{l}}\to X\right)}{\inf}E\left[\ell_{X}\left(f_{x}\left(Z_{l}^{\left(i\right)}\right);X\right)\right] \end{array}$$

where $f_{x}\left(\cdot \right):R^{n_{l}}\to X$ is an estimator living in the function space $C(R^{n_{l}}\to X)$ and $\ell_{X}\left(\cdot ;\cdot \right)$ is a loss function. We will take the loss function to correspond to the squared error so that the Z-X measure reduces to the well-known minimum mean-squared error given by:

(4) $$\begin{array}{cc} m_{Z_{l}^{\left(i\right)};X} & =\mathrm{mmse}\left(X|Z_{l}^{\left(i\right)}\right)=E\left[{\left(X-E\left[X|Z_{l}^{\left(i\right)}\right]\right)}^{2}\right] \end{array}$$

where the function $f_{x}\left(\cdot \right)$ that minimizes the right-hand side of Equation (3) is the well-known conditional mean estimator. Our rationale for adopting this quantity to capture the relationship between the network representation and the data in *lieu* of mutual information—which is used in the conventional IB—is manifold:

- First, the minimum mean-squared error can act as a proxy to capture fitting—the lower the MMSE, the easier it is to recover the data from the representation—and compression—the higher the MMSE, the more difficult it is to estimate the data from the representation.
- Second, this quantity is also easier to estimate than mutual information, allowing us to capture the phenomena above reliably (see Section 5.1).
- Finally, the minimum mean-squared error is also connected to mutual information (see Section 3.1).

**Z-Y measure:** Our second quantity describes the difficulty in recovering the original label *Y* from some intermediate network representation $Z_{l}^{\left(i\right)}$ as follows:

(5) $$\begin{array}{cc} m_{Z_{l}^{\left(i\right)};Y} & =\underset{f_{y}\in C\left(R^{n_{l}}\to Y\right)}{\inf}E\left[\ell_{Y}\left(f_{y}\left((Z_{l}^{\left(i\right)}\right);Y\right)\right] \end{array}$$

where $f_{y}\left(\cdot \right):R^{n_{l}}\to Y$ is an estimator living in the function space $C(R^{n_{l}}\to Y)$ and $\ell_{Y}\left(\cdot ;\cdot \right)$ is a loss function. We will take the loss function to correspond to the cross-entropy so that the Z-Y measure reduces to the well-known conditional entropy given by:

(6) $$\begin{array}{c} m_{Z_{l}^{\left(i\right)};Y}=H\left(Y|Z_{l}^{\left(i\right)}\right) \end{array}$$

where the function $f_{y}\left(\cdot \right)$ that minimizes the right-hand side of Equation (5) should model the distribution of the label given the representation. We also adopted this measure because it connects directly to performance—hence the ability of the network to generalize—but also to mutual information (see Section 3.1).

**Plane and Dynamics of the Z-X and Z-Y Measures:** Equipped with the measures in Equations (4) and (6), one can immediately construct a two-dimensional plane plotting the Z-X measure $m_{Z_{l}^{\left(i\right)};X}$ against the Z-Y measure $m_{Z_{l}^{\left(i\right)};Y}$ as a function of the number of network training epochs i=1,2,3,… in order to understand (empirically) how a particular neural network operates. Such a plane and the associated dynamics are the analogue of the IB plane and the IB dynamics introduced in [8].

### 3.1. Connecting our Approach to the Information Bottleneck

Our approach is also intimately connected to the conventional information bottleneck because—as alluded to earlier—our adopted measures are also connected to mutual information. First, in accordance to [51] (Theorem 10), we can bound the mutual information between the data *X* and the representation $Z_{l}^{\left(i\right)}$ as follows:

(7) $$\begin{array}{cc} \frac{1}{2}I\left(X;Z_{l}^{\left(i\right)}\right) & \geq \mathrm{var}\left(X\right)-\mathrm{mmse}\left(X|Z_{l}^{\left(i\right)}\right) \end{array}$$

where var(·) represents the variance of the random variable.

Second, we can also trivially express the mutual information between the data *Y* and the representation $Z_{l}^{\left(i\right)}$ as follows:

(8) $$\begin{array}{cc} I(Z_{l}^{\left(i\right)};Y) & =H\left(Y\right)-H\left(Y|Z_{l}^{\left(i\right)}\right) \end{array}$$

However, the main advantage of our approach in relation to the traditional IB is that it is much easier to estimate the proposed Z-X and Z-Y measures than the corresponding mutual information in high-dimensional settings; see Section 5.1.

## 4. Implementation Aspects

### 4.1. Experimental Procedure

The crux of our approach involves tracking how the Z-X and Z-Y measures evolve during the network optimization process as a function of the learning algorithm epochs. However, we cannot estimate the measures in Equations (4) and (6) directly because we do not have access to the relevant probability distributions. Instead, we will leverage the variational representations of the Z-X measure in Equation (3) and the Z-Y measure in Equation (5) to approximate the measures in a data-driven manner, given access to a dataset $S={\left\{X(k),Y(k)\right\}}_{k=1}^{n}$ consisting of various input–label pairs.

In particular, given this dataset $S={\left\{X(k),Y(k)\right\}}_{k=1}^{n}$, we used learnable functions $f_{\phi }:R^{n_{l}}\to X$ and $f_{\psi }:R^{n_{l}}\to Y$—which are neural networks parameterized by ϕ and ψ, respectively—to approximate the measures in Equations (4) and (6) as follows:

(9) $$\begin{array}{cc} m_{Z_{l}^{\left(i\right)};X} & =\underset{f_{x}}{\inf}E\left[\ell_{x}\left(f_{x}\left(Z_{l}^{\left(i\right)}\right);X\right)\right]\leq \min_{\phi }E\left[\ell_{x}\left(f_{\phi }\left(Z_{l}^{\left(i\right)}\right);X\right)\right]\approx \min_{\phi }\frac{1}{n}\sum_{k=1}^{n}\ell_{x}\left(f_{\phi }\left(Z_{l}^{\left(i\right)}\left(k\right)\right);X\left(k\right)\right)\\ \; & ={\hat{m}}_{Z_{l}^{\left(i\right)};X} \end{array}$$

and

(10) $$\begin{array}{cc} m_{Z_{l}^{\left(i\right)};Y} & =\underset{f_{y}}{\inf}E\left[\ell_{y}\left(f_{y}\left(Z_{l}^{\left(i\right)}\right);Y\right)\right]\leq \min_{\psi }E\left[\ell_{y}\left(f_{\psi }\left(Z_{l}^{\left(i\right)}\right);Y\right)\right]\approx \min_{\psi }\frac{1}{n}\sum_{k=1}^{n}\ell_{y}\left(f_{\psi }\left(Z_{l}^{\left(i\right)}\left(k\right)\right);Y\left(k\right)\right)\\ \; & ={\hat{m}}_{Z_{l}^{\left(i\right)};X} \end{array}$$

respectively, where the learnable function parameters ϕ and ψ are drawn from Φ and Ψ, $Z_{l}^{\left(i\right)}=f_{\theta_{l}}^{\left(l\right)}\left(Z_{l-1}^{\left(i\right)}\right)$, and $Z_{l}^{\left(i\right)}\left(k\right)=f_{\theta_{l}}^{\left(l\right)}\left(Z_{l-1}^{\left(i\right)}\left(k\right)\right)$. Note that—in view of the fact that $\left\{f_{\phi }:\phi \in \Phi \right\}\subset C\left(R^{n_{l}}\to X\right)$ and $\left\{f_{\psi }:\psi \in \Psi \right\}\subset C\left(R^{n_{l}}\to Y\right)$—one is confronted with an immediate trade-off: the higher the number of parameters in the learnable functions, the closer the upper bounds to the measures in Equations (9) and (10) are to the actual measure but also the higher the number of samples that may be required to approximate the upper bound reliably. This will be further discussed in Section 5.1.

**Figure 1:**
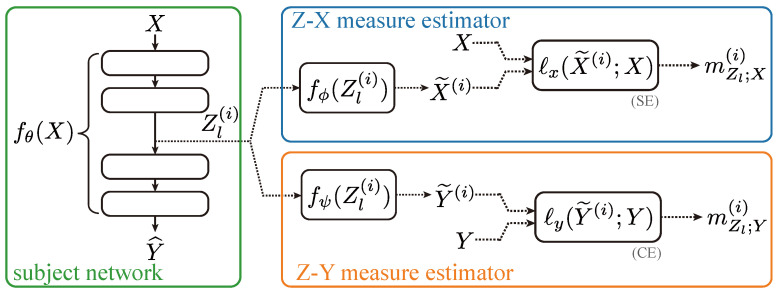
Proposed approach. We used two estimator neural networks $f_{\phi }\left(\cdot \right)$ and $f_{\psi }\left(\cdot \right)$ to study the behavior of the Z-X measure and the Z-Y measure associated with the different representations of the subject network $f_{\theta }\left(\cdot \right)$. The $\ell_{x}\left(\cdot \right)$ and $\ell_{y}\left(\cdot \right)$ are squared loss and cross-entropy, respectively.

**Figure 2:**
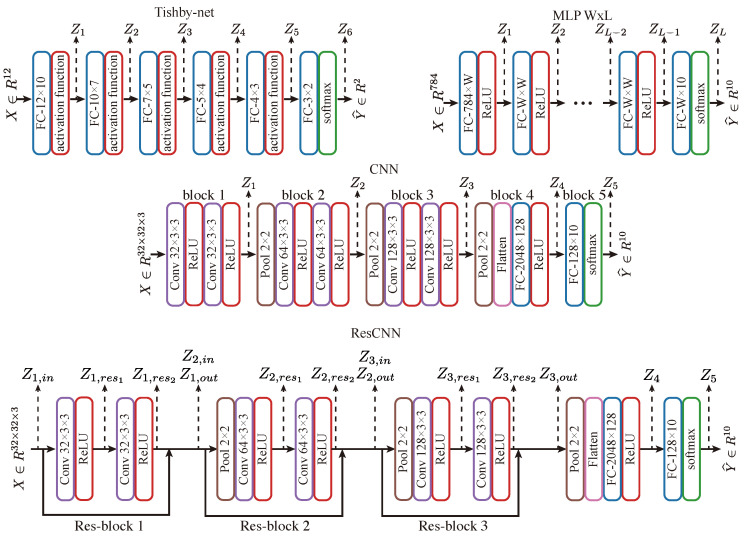
The architectures of subject neural networks involved in this paper. Tishby-net will be trained on the Tishby-dataset proposed in [8], MLP W × L will be trained by MNIST dataset [53], and CNN and ResCNN will be trained on CIFAR-10 dataset [55]. FC stands for fully connected layer, Conv represents the convolutional layer, and Pool refers to the max pooling layer. Note that we intentionally kept the architecture of the CNN as close to ResCNN as possible to enable a better-controlled comparison in later experiments.

**Figure 3:**
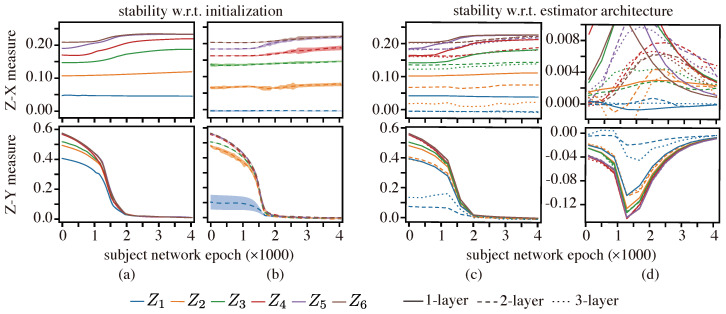
Z-X and Z-Y measures estimate on the Tishby-net: (**a**,**b**) stability with regard to the initialization of estimator networks, and (**c**,**d**) stability with regard to the architecture of estimator networks. The lines are averaged over five different initializations, and the shadow is *five times* the standard deviation. The representations (e.g., $Z_{1}$) are taken from the corresponding layer of the Tishby-net in Figure 2. The measures in (**a**) are estimated with 1-layer estimators with varying initializations, and measures in (**b**) are estimated with 2-layer estimators with different initializations. (**c**) compares the measures estimated by estimators with different depths, while the curves in (**d**) depict the measures increasing/decreasing trend, obtained by taking the derivative of (**c**).

Our setup is summarized in Figure 1. We re-emphasize that there were three neural networks involved in our study: (1) $f_{\theta }\left(\cdot \right)$ is the network whose dynamics we wish to study (in the green box of Figure 1), (2) $f_{\phi }\left(\cdot \right)$ represents the neural network used to approximate the Z-X measure (in the blue box of Figure 1), and (3) $f_{\psi }\left(\cdot \right)$ represents the neural network used to estimate the Z-Y measure (in the yellow box of Figure 1)

We optimized these networks using the procedure outlined in Algorithms 1 and 2. The algorithm used to optimize the neural network $f_{\theta }$ can be used with different neural network models, different learning algorithms, or different datasets. Note that this algorithm saves the neural network learnable parameters as checkpoints every several epochs (as shown in Algorithm 1), where we used *T* to control the total number of checkpoints to limit computational overhead.

In turn, the algorithm used to train the estimator networks $f_{\phi }\left(\cdot \right)$ and $f_{\psi }\left(\cdot \right)$ uses the Adam optimizer with a learning rate of 0.01 for efficient and stable estimation. The estimator networks were initialized using the standard Xavier [52] initialization (unless otherwise specified), and the estimator networks were also optimized until convergence (which is identified by the increase in loss value on the validation set).

We note that we trained the subject network on a training set, but we trained the estimator networks on a different (independent) validation set in order to obtain estimates of the Z-X and Z-Y measures that can also capture generalization behavior. The Tishby-dataset is an exception since it does not have a separate validation set. Note, however, that, in the IB literature, few studies have reported differences in trends by estimating the relevant mutual information quantities on the training set or an independent validation set. Some studies (e.g., [8]) also do not specify the dataset used to compute the mutual information measures.

We will be referring for simplicity in the sequel to the network whose dynamics we wish to study (i.e., $f_{\theta }\left(\cdot \right)$) as the *subject network* and to the networks whose purpose is to estimate the relevant measures (i.e., $f_{\phi }\left(\cdot \right)$ and $f_{\psi }\left(\cdot \right)$) as the *estimator networks*.    
**Algorithm** **1:** Train the subject network
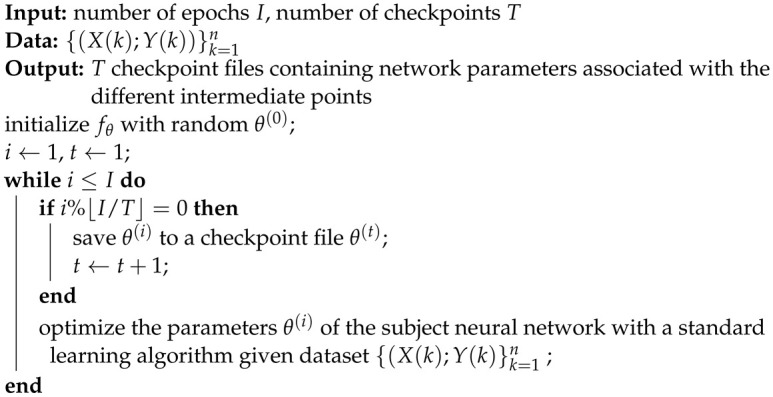


**Algorithm** **2:** Estimate Z-X measure and Z-Y measure

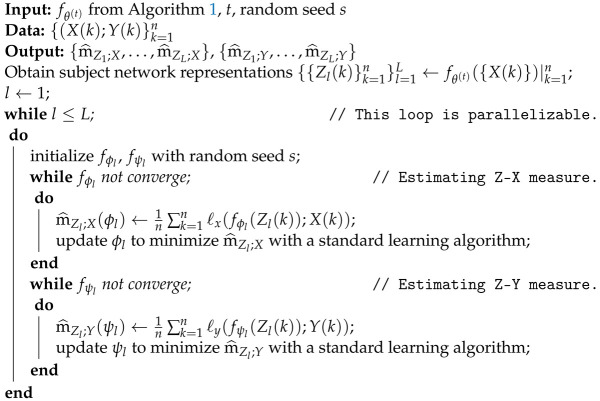



### 4.2. Experimental Setups

Our experiments studied the effect of the (subject) network model architecture, the (subject) network learning algorithm, and the dataset on key aspects, such as network fitting, compression, and generalization, via the Z-X and the Z-Y dynamics; see also Table 1. We therefore summarize next the main models, learning algorithms, and datasets used in the study reported in Section 5.

**Subject Network Models:** We adopted a series of neural network models, including: (1) the Tishby-net, proposed by [8], with the Tishby-dataset, consisting of 4096 samples with binary labels; (2) MLP models with varying number of layers and varying width per layer with an MNIST dataset [53], which has 60,000 grayscale handwritten digit images for training and 10,000 for validation; (3) a convolutional neural network (CNN) with VGG-like [54] architecture trained on the CIFAR-10 [55] and CINIC [56] dataset, where the CIFAR-10 dataset comprises 50,000 RGB images categorized into 10 classes for training and 10,000 for validation, and the CINIC dataset consists of 900,000 training samples labeled in the same way as the CIFAR-10 dataset; and (4) a ResCNN model on the CIFAR-10 dataset, which is a CNN architecture with residual connections modified from the original CNN. The various models and datasets will allow us to study the effect of model architectures and datasets on network dynamics. These models are illustrated in Figure 2. Note that, for MNIST and CIFAR-10 datasets, we separated the validation sets into two halves of the same size: one half was used for plotting the dynamics, and the other half served as the test set for evaluating generalization performance.

**Subject Network Learning Algorithm:** We also adopted a series of learning algorithms, including (1) training a CNN on the CIFAR-10 dataset using different optimizers, such as non-adaptive (SGD, SGD-momentum) and adaptive (RMSprop, Adam); (2) training an MLP on the MNIST dataset with or without weight decay regularization, where the regularization hyper-parameter was set to 0.001; and (3) training a CNN on the CIFAR-10 dataset with or without dropout regularization, where the dropout was only applied on the fully connected layer of the CNN as implemented in [54]. These setups allowed us to study the effect of different optimization algorithms and regularization methods on network dynamics.

**Estimator Neural Network Model and Algorithms:** We deployed a variety of estimator network architectures that depend on the architecture of the subject network (namely, the specific shape of the subject network representations in the different layers) as follows:

- For Tishby-net and MLP WxL models, the models for both the Z-X measure estimator and Z-Y measure estimator are fully connected neural networks. The input layer of the estimator networks matches the dimension of the representation ($Z_{l}$), while the output layer has a dimension equivalent to either the input vector (for Z-X measures) or label length (for Z-Y measures). If the estimator network has multiple layers, its hidden layers will be connected using ReLU non-linearity and have a number of neurons equal to the dimension of representation ($Z_{l}$).
- To estimate the Z-Y measure for CNN and ResCNN, we flattened the representation into a vector and employed the same network architecture as for the Z-Y measure estimator of Tishby-net and MLP WxL models. In turn, to estimate the Z-X measure, we used a convolution layer with a 3 × 3 kernel size to map the representation into the input space of 32×32×3. However, if the representation is down-sampled by a pooling layer (e.g., Figure 2 CNN $Z_{2}$), we up-sampled it using a transposed convolutional layer with a 2 × 2 kernel size before feeding it into the convolutional layer. The number of transposed convolutional layers equals the number of pooling layers that the representation has gone through since each transposed convolutional layer can only up-sample the representation by a factor of 2. ReLU non-linearity exists between all hidden layers. For example, when the representation is generated by a layer with two pooling layers before it (e.g., Figure 2 CNN $Z_{3}$), the estimator for the Z-X measure would contain two transposed convolutional layers.

These estimators have been shown to be computationally efficient, offering stable results.

### 4.3. Other Practical Considerations

In view of the fact that we computed the relevant measures for different layers of the subject network at different learning epochs, we also adopted various other practical tricks to improve the computation efficiency as follows:

(1) **Parallelize checkpoint enumeration t∈{1,2,⋯,T}:** To plot the Z-X / Z-Y measures dynamics, we need to calculate these quantities at different checkpoints saved from various epochs during the training of the subject network.
  We can easily deploy multiple Algorithm 2 instances on different checkpoints saved per Algorithm 1 in parallel;
(2) **Parallelize layer iteration l∈{1,2,⋯,L}:** We can also break up the iteration of *l* layers in Algorithm 2 into parallel processes since the estimations of the measures on different layers are independent;
(3) **Parallelize estimation of Z-X measure and Z-Y measure:** We can also deploy the Z-X measure estimator and the Z-Y measure estimator on different processes because they are also independent;
(4) **Warm-start:** Moreover, we can accelerate the convergence of estimator networks by using warm-start. We randomly initialized and trained the estimators from scratch in the first checkpoint for Tishby-net and MLP WxL models. We then used the learned parameters as initialization for the estimators in subsequent checkpoints. However, we did not use warm-start in CNN and ResCNN estimator networks as it does not noticeably accelerate convergence in these cases.

We deployed our algorithms on a server equipped with one NVIDIA Tesla V100 GPU.

## 5. Results

We now build upon the proposed framework to explore the dynamics of the Z-X and Z-Y measures and their relationship with fitting/compression (F/C) phases and generalization in a range of neural network models. In particular, the fitting phase refers to the initial phase of training where the Z-X measure decreases with the number of epochs, indicating that the network is attempting to fit the dataset. This phase commonly occurs during early training. On the other hand, the compression phases refer to the subsequent increase in the Z-X measure, indicating the compression of information in the network.

Firstly, we experimentally examined whether the estimation of the proposed measures is stable. Then, we examined the impact of (1) the model architecture; (2) the learning algorithm including optimizer and regularization techniques; and (3) the data on the dynamics of the measures.

The results will be presented using Z-X and/or Z-Y dynamics, and the tables show the losses, accuracy, and generalization error of each experiment. In the figures, the x-axes or y-axes will be shared unless specified otherwise by the presence of ticks.

### 5.1. Z-X and Z-Y Measures Estimation Stability

The reliability of the estimation of the proposed measures is critical for extracting robust conclusions about the behavior of the Z-X and Z-Y dynamics in a neural network. Such studies are, however, largely absent in the information bottleneck literature [12].

#### 5.1.1. Criteria to Describe the Stability of Estimated Measures

We assessed the stability of the Z-X and Z-Y measures estimation using two criteria:

- **Stability with regard to the initialization of estimator networks:** First, we explored how different initializations of an estimator network affect the Z-X and Z-Y measures.
- **Stability with regard to the architecture of estimator networks:** Second, we also explored how (estimator) neural network architectures—with different depths—affect the estimation of the Z-X and Z-Y measures.

#### 5.1.2. Subject Networks, Estimator Networks, and Datasets Involved

We examined the stability of Z-X and Z-Y measures estimates in both fully connected and convolutional subject networks. In particular, we used: (1) a Tishby-net (which has an MLP-like architecture) trained on the Tishby-dataset classification task with a standard stochastic gradient descent (SGD) optimizer, and (2) a CNN trained on the CIFAR-10 classification task trained with an Adam optimizer. However, we noticed that the Tishby-net may not always converge due to its simple architecture and small dataset size of 4096 samples. Therefore, we repeated the training process multiple times with different initializations and only retained converged subject networks to ensure meaningful results. We built estimator networks as elaborated in the previous sections, and their architectures are detailed in Appendix A.

To verify the first stability criterion, we tested different initializations by modifying the random seed of the Xavier initializer. For the second stability criterion, we experimented with estimators at different depths.

#### 5.1.3. Are the Measures Stable in the MLP-like Subject Neural Networks?

Figure 3 depicts the Z-X and Z-Y measures estimates on the Tishby-net. Specifically, panels (a) and (b) display the behavior of such measures under different initializations of a one-layer and two-layer estimator network, respectively. Our results indicate that these measures are robust to changes in the initialization of the estimator network (for a given estimator network architecture).

In turn, panels (c) and (d) depict the behavior of the Z-X and Z-Y measure estimates for different estimator network architectures. It is clear that the capacity of the estimator (which depends on the number of estimator network layers) may affect the exact value of the Z-X and Z-Y measures estimate, indicating the presence of a bias; however, such estimators can still capture consistent trends (such as increases and decreases in the measures that are critical to identifying fitting or compression behavior; see panel (d)).

We however note—as we had elaborated previously—that the estimator networks need to be sufficiently complex to emulate a conditional mean estimator—to estimate the Z-X measure—or to emulate the conditional distribution of the label given the representation—to estimate the Z-Y measure. This may not always be possible depending on the complexity/capacity of the estimator network e.g., one-layer estimator networks are only capable of representing linear estimators whereas two-layer networks can represent more complex estimators (therefore, linear one-layer networks cannot reliably estimate the minimum mean-squared error unless the random variables are Gaussian). However, our results suggest that, with a two-layer network, we may already obtain a reliable estimate since—except for some representations—the difference in the measures estimated using a two-layer network does not differ much from those using a three-layer network. Naturally, with an increase in the capacity of the estimator networks, one may also need additional data in order to optimize the estimator network to deliver a reliable network, but our results also suggest that the variance of the estimates is relatively low for both two-layer and three-layer estimators. Further, the results in [57] suggest that the difference between the estimated value and the true value for our Z-X measure decays rapidly with the number of points in the (validation) dataset (note, however, that these results only apply for scalar random variables). Therefore, we will adopt a two-layer estimator network in our study of MLPs in the sequel.

We conducted a more robust analysis of the efficacy of different estimators using a Gaussian mixture data model in Appendix B, where we can also directly analytically compute the mean-squared error for comparison purposes.

#### 5.1.4. Are the Measures Stable in the Convolutional Subject Neural Networks?

**Figure 4:**
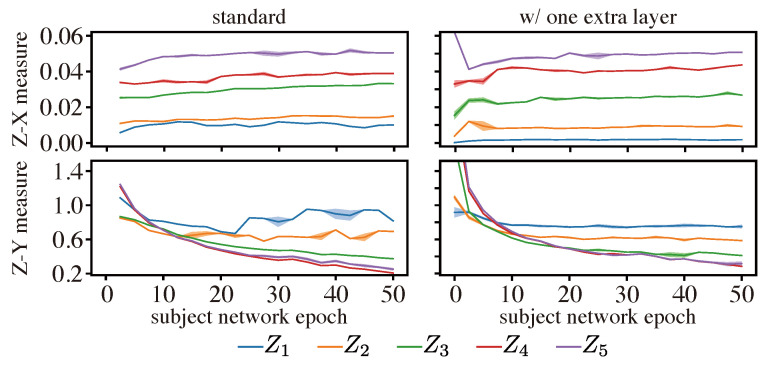
Z-X and Z-Y measures estimate on the CNN: the lines are averaged over five different initializations, and the shadow is *five times* the standard deviation. The representations (e.g., $Z_{1}$) are taken from the corresponding layer of the CNN in Figure 2. Note that the violation of the data processing inequality (DPI) observed in the Z-Y measure is attributed to the use of a pre-defined estimator model. This aspect is also acknowledged in the context of the V-information framework, as discussed in [38].

Figure 4 shows the Z-X and Z-Y measure estimates on the CNN. To test the stability criteria, we again used different estimator network initializations (varying the random seed of the Xavier initializer) and different estimator network architectures. We first plotted the Z-X dynamics and Z-Y dynamics based on the setup described in Section 4, and the results are shown in the left column of Figure 4. Then, for comparison, we added an extra convolutional layer to all Z-X estimators and a fully connected layer to all Z-Y estimators, and the results are displayed in the right column of Figure 4.

**Figure A3:**
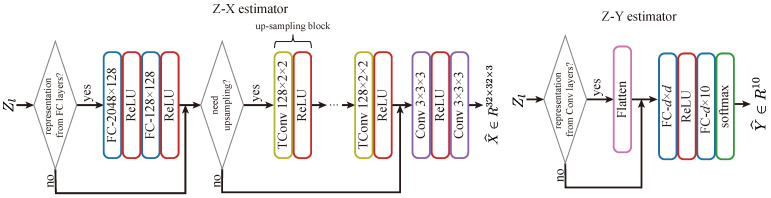
Architectures of the Z-X and Z-Y estimator networks for CNN and ResNet. The number of up-sampling blocks in the Z-X estimator is set to be equal to the number of down-sampling blocks in the network being analyzed, and the dimension of the fully connected layer *d* in the Z-Y measure is determined by the shape of the flattened input vector length. To test the stability of the estimators at different depths (as described in Section 5.1), we added an extra convolutional layer with ReLU non-linearity at the end of the Z-X estimator, and a fully connected layer with a width of *d* before the first fully connected layer in the Z-Y estimator.

The results show that both estimator networks lead to relatively consistent and stable measure estimates. This suggests that our proposed measures can be reliably inferred using such estimator networks—under different initializations—even in this high-dimensional setting that poses significant challenges to mutual information estimators. Comparing the dynamics estimated by the standard estimator architecture and the one with an extra layer, we observed that the trends of the dynamics are similar. Hence, we used the standard setup in the rest of the paper due to its higher computational efficiency, which is illustrated in Figure A3.

We next relied on this approach to estimate the Z-X and the Z-Y dynamics for different (subject) neural network models and algorithms in order to cast further insights into the compression, fitting, and generalization dynamics of deep learning.

### 5.2. The Impact of Model Architectures to the Network Dynamics

We started our study by investigating the effect of the neural network model on the Z-X and Z-Y dynamics of neural networks. We considered both MLPs with different activation functions, depths, and widths. We also considered CNN and res-net architectures. Our study will allow us to identify possible fitting, compression, and generalization behavior.

#### 5.2.1. Does the Activation Function Affect the Existence of F/C Phases?

We began by examining whether the presence of the fitting and compression (F/C) phases is dependent on the activation function used in the network. This topic has been explored in previous studies using the IB approach [8,11,24,27], but different studies have led to different conclusions [27].

**Setups:** We deployed Tishby-net architecture with various activation functions, including both saturating (tanh and softsign [58]) and non-saturating (ReLU [59], ELU [60], GELU [58], swish [61,62], PELU [63], and leaky-ReLU [64]) options. The Tishby-net was trained on the Tishby-dataset using the same optimizer and hyper-parameter setups as described in the literature [8,24]. The Z-X and Z-Y measures were estimated using two-layer estimators, as argued in Section 5.1.

**Figure 5:**
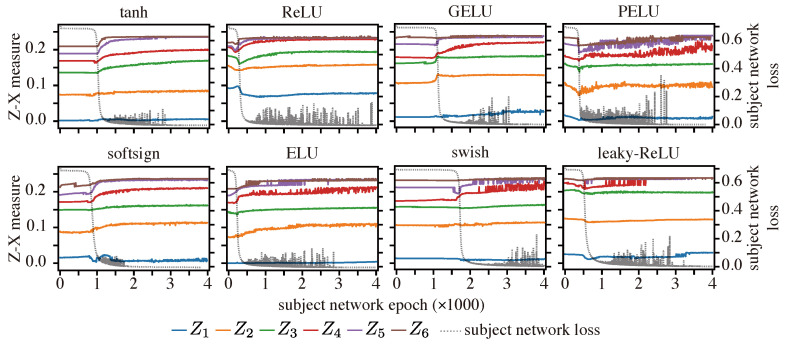
Z-X dynamics on Tishby-net with different activation functions. The left y-axes displays the Z-X measure estimate values, while the right y-axes represent the cross-entropy loss value of the subject network.

**Results:** Figure 5 reveals that the Z-X dynamics exhibit a consistent pattern among all Tishby-nets, characterized by an initial decrease in Z-X measures followed by an increase. Note that the initial decrease happens prior to the decrease in the subject network loss. There can be a longer period of epochs where the network struggles to converge and, during this phase, the changes in the Z-X measure may not be easily visible. The Z-X dynamics in some experimental setups, such as PELU, display fluctuation, which we attribute to the unstable convergence of the subject network, as evidenced by the fluctuations in the subject network loss. Moreover, the increases in Z-X measures coincide with epochs where the network experiences a decrease in loss. These observations suggest that the F/C phases are likely to occur in the network, regardless of the activation function employed. Our observation is in line with some of the previous studies that have used MI measures, such as [8,11].

#### 5.2.2. How Do the Width and Depth of an MLP Impact Network Dynamics?

We now examine the effect of the MLP width (number of neurons per layer) and depth on the Z-X and Z-Y dynamics.

**Setups:** For the MLP width analysis, we constructed four-layer MLPs with different numbers of neurons per layer: 16, 64, and 512. For the MLP depth experiment, we fixed the width of the subject network to 64 and varied its depth from two to six hidden layers. All models were trained on the full MNIST dataset using a standard SGD optimizer with a fixed learning rate of 0.001. We also used two-layer estimator networks to estimate the Z-X and Z-Y measures.

**Figure 6:**
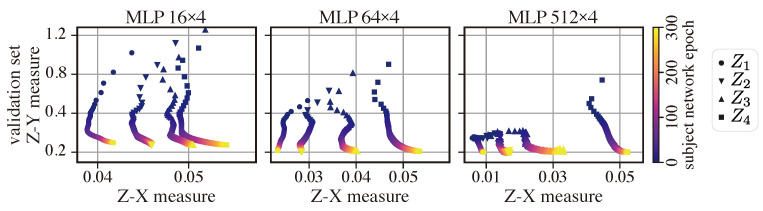
Z-X/Z-Y measures dynamics plane of MLP networks with different widths. The representations (e.g., $Z_{1}$) are taken from the corresponding layer of the MLP WxL network in Figure 2.

Figure 6 depicts the dynamics of the Z-X measure against the Z-Y measure for MLP networks with four layers and with different widths. As shown in Table 2, the best generalization performance is associated with the model MLP 512 × 4. We can observe that all MLP networks exhibit fitting and compression phases. However, wider networks (e.g., MLP 512 × 4) tend to begin compressing earlier, while the thinner ones (e.g., MLP 16 × 4) tend to have a longer fitting phase. This trend suggests that wider networks are able to fit data more quickly. We can also observe that the networks with more neurons per layer (MLP 512 × 4) exhibit more compression than network with fewer neurons per layer (MLP 16 × 4). Interestingly, the MLP 512 × 4 model also exhibits the best generalization performance, so one can potentially infer that significant compression may be necessary for good generalization [8,29].

**Figure 7:**
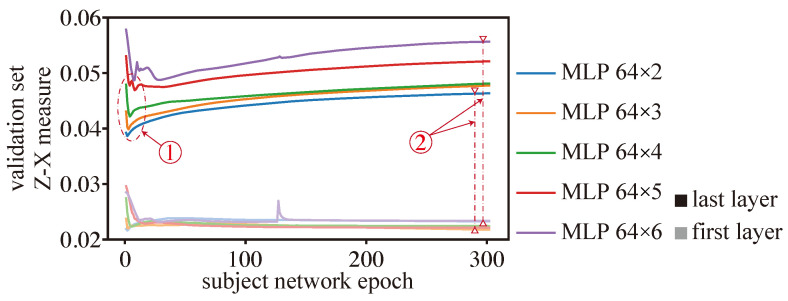
Z-X dynamics of the MLP 64 networks with different depths. The curves with higher saturation correspond to the last layer of the MLP model, while those with lower saturation belong to the first MLP layer.

Figure 7 depicts the dynamics of the Z-X measure (associated with the first and last layers) of MLPs with a width of 64 and with different depths (we note that the best generalization performance is associated with the model MLP 64 × 3). In terms of fitting, we can observe that the different MLPs experience a fitting phase. However, deeper models such as MLP 64 × 5 and MLP 64 × 6 appear to experience a more pronounced fitting phase than shallower models, though deeper models still exhibit a higher Z-X measure than shallower ones toward the end of this fitting phase (see marker #1). In terms of compression, we find that deeper networks (e.g., MLP 64 × 5, MLP 64 × 6) compress data more aggressively than shallower ones. Indeed, the gap between the Z-X measure value between the last layer and the first layer of the network is much higher for a deeper model than for shallower ones (as indicated by marker #2).

We also highlight that the MLP 64×3 network, which demonstrated the best generalization performance (refer to Table 2), exhibited a significant fitting phase similar to MLP 64 × 2, as well as a notable compression phase close to MLP 64-4.

Overall, shallow networks may have difficulty compressing data effectively, while the layers close to the output in the deep networks may lose important information and cannot fit data well. We hypothesize that both of these phenomena—which are both present in the MLP 64×3 network—can have an impact on a network’s ability to generalize effectively.

#### 5.2.3. How Do the Number of Kernels and Kernel Size of a CNN Impact Network Dynamics?

We now examine the effect of the kernels, including their number and size, on the Z-X and Z-Y dynamics in a CNN.

**Setups:** To analyze the impact of the number of kernels on network F/C phases in CNNs, we adjusted the number of kernels by a factor derived from the baseline CNN architecture shown in Figure 2. To analyze the impact of the kernel size, we used 1 × 1, 3 × 3 (baseline), 5 × 5, and 7 × 7 kernel sizes for all convolutional layers The CNN models were trained on the CIFAR-10 dataset using the Adam optimizer with a learning rate of 0.001. We utilized minimal estimator networks, as described in the previous section.

**Figure 8:**
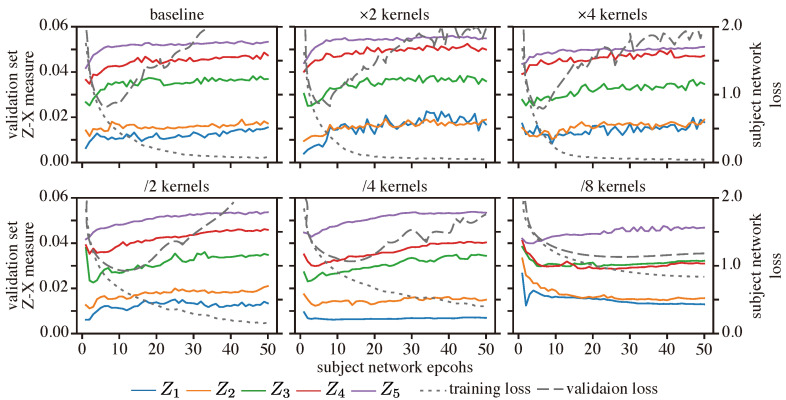
Z-X dynamics of the CNN network with different number of kernels on each layer. We make modifications based on the baseline CNN structure shown in Figure 2. For example, “×2” means doubling the number of kernels in each convolutional layer, while “/2” means halving the number of kernels in each convolutional layer. The representations (e.g., $Z_{1}$) are taken from the corresponding block of the CNN network in Figure 2.

**Results:** Figure 8 depicts the Z-X dynamics of our CNN network with different numbers of kernels. We observe that having a low number of kernels (e.g., /4, /8) seems to impair both the fitting and compression process, particularly in early layers (e.g., layers 1 and 2). In contrast, we observed that a high number of kernels do not significantly impact the F/C phases or the generalization performance. Indeed, as shown in Table 3, CNNs with more kernels (e.g., ×2, ×4) have a similar test loss performance to the baseline model (note that the best test loss performance corresponds to the ×4 model, and that its generalization performance is also similar to that of the baseline model). This suggests that adding more kernels to a well-generalized CNN may not significantly impact the F/C phases and may not lead to an improved generalization.

**Figure 9:**
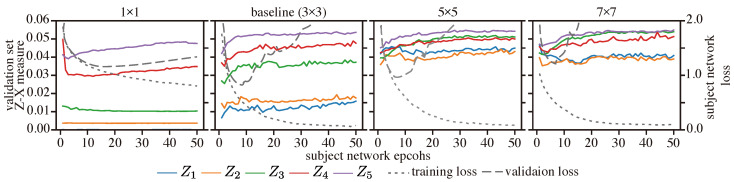
Z-X dynamics of the CNN network with different kernel sizes. The representations (e.g., $Z_{1}$) are taken from the corresponding block of the CNN network in Figure 2.

Figure 9 depicts the Z-X dynamics of our CNN network with different kernel sizes. It appears that networks with large kernels fail to fit and compress, but networks with small kernels also exhibit little fitting and compression. Indeed, the best test loss and generalization performance are associated with the CNN model with a 3 × 3 kernel size, which also exhibits a more pronounced fitting and compression phase (refer to Table 3).

Overall, we hypothesize that selecting an appropriate kernel size can improve a network’s ability to both fit and compress data, leading to a better generalization performance, which is in line with the conclusion in [8,29].

#### 5.2.4. How Does Residual Connection Affect the Network Dynamics?

We finally assessed the impact of residual connections—introduced in [65]—on neural network learning dynamics, since these have been frequently used to address the gradient vanishing problem in very deep neural networks. We note that some works [13,18] have studied the behavior of ResNet or DenseNet (which also contain residual connections [66]). However, these studies did not delve into how residual connections may impact the information bottleneck of hidden layer representations and their relation to generalization.

**Setup:** We deployed a ResCNN, as elaborated in the previous section, that was trained using an Adam optimizer with a learning rate of 0.001 on the CIFAR-10 dataset. We also used the standard estimator network setups elaborated in Section 4.2 and shown in Appendix A Figure A3.

**Figure 10:**
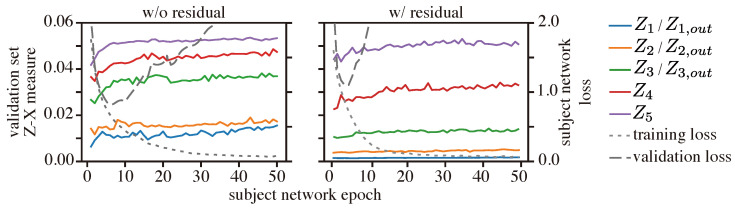
Z-X dynamics of CNNs with or without residual connections. The representations (e.g., $Z_{1}$, $Z_{1,out}$) are taken from the corresponding locations of the CNN or ResCNN network shown in Figure 2.

**Results:** We first analyzed the behavior of the Z-X dynamics at the output of the residual blocks (e.g., $Z_{1,out}$) and the fully connected layers, and compared it with the CNN with a similar architecture but without residual connections; see Figure 10.

We notice that the ResCNN tends to have less pronounced compression in the (residual) convolutional blocks, e.g., the Z-X dynamic of $Z_{3}$ (without residual connection) shows a more pronounced increase than that of $Z_{3,out}$ (with residual connection). Additionally, we can see that the model with residual connection depends more on the fully connected layers to compress the Z-X measure, which is demonstrated by the significantly wider gap between representations $Z_{4}$ and $Z_{5}$, as well as between $Z_{4}$ and $Z_{3}/Z_{3,out}$ in the residual model.

**Figure 11:**
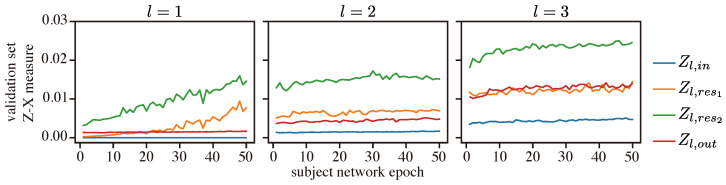
Z-X dynamics of the ResCNN in each residual block. *l* is the index of the residual block. The representations (e.g., $Z_{1,in}$) are taken from the corresponding block of the ResCNN network in Figure 2.

We then inspected the behavior of the Z-X measure and the Z-Y measure within each residual block; see Figure 11 (note that the dynamics of the Z-X and Z-Y measures associated with $Z_{1,in}$ are flat because $Z_{1,in}$ corresponds to *X*).

We can observe that, within each residual block (i.e., for a given index *l*), the Z-X measure of $Z_{l,out}$ is generally lower than that of $Z_{l,res_{1}}$ and $Z_{l,res_{2}}$. This is because the representation $Z_{l,out}$ is the sum of $Z_{l,res_{2}}$ and $Z_{l,in}$ and thus retains more information associated with the data.

We can also observe that, in every residual block, the Z-X dynamics of $Z_{l,res_{1}}$ and $Z_{l,res_{2}}$ have a pronounced increase over the epoch, while the Z-X dynamics of $Z_{l,in}$ and $Z_{l,out}$ are relatively stable. This suggests that each residual block may learn to form a mini-bottleneck. However, the overall network does not exhibit a visible compressing phase when observing the output of the residual blocks alone. Our experiments demonstrate the distinct behavior of networks with residual connections compared to those without.

### 5.3. The Impact of Training Algorithm to the Network Dynamics

A neural network generalization ability also tends to depend on the training procedure, including the learning algorithm and regularizers. Therefore, we now explore how different learning settings affect neural network Z-X and Z-Y measures dynamics.

#### 5.3.1. How Does the Optimizer Impact the Network Dynamics?

It was suggested by [29] that the Adam optimizer leads to a better performance during the fitting phase, but it tends to perform worse during the compression phase. We investigated, under the lens of our approach, the effect of Adam and various other optimizers on neural network learning dynamics.

**Setup:** Our experiments were conducted on CNNs (with the standard architecture illustrated in Figure 2) trained on the CIFAR-10 dataset using different optimizers. Specifically, we experimented with non-adaptive optimizers such as SGD and SGD-momentum [67], as well as adaptive optimizers such as RMSprop [68]. We also considered the Adam optimizer [69], which can be viewed as a combination of a momentum optimizer and RMSprop optimizer, representing a hybrid approach. We used standard hyper-parameters commonly used for CIFAR-10 classification tasks, setting the learning rate to 0.001 for all optimizers and a momentum parameter of 0.9 (if applicable). Our estimator networks are akin to those used in previous studies.

**Figure 12:**
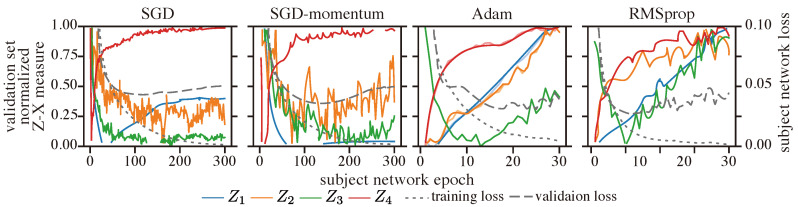
Z-X dynamics for a CNN trained with different optimizers. The representations (e.g., $Z_{1}$) are taken from the corresponding block of the CNN network in Figure 2.

**Results:** Figure 12 shows the behavior of the normalized Z-X measure for CNNs trained with different optimizers. We normalized this measure using min-max normalization to allow for a better visualization of relative changes in performance. Specifically, each Z-X dynamic curve was normalized individually, and the minimum and maximum values were taken from the curve after the 50th epoch, as we observed that all Z-X dynamics enter the compression phase before this epoch.

We observe that SGD and SGD-momentum exhibit similar fitting phases, while Adam and RMSprop also display similar fitting phases. We can also note that, when trained on the Adam and RMSprop optimizer—which are adaptive optimizers—the representations associated with the various layers exhibit major compression; in contrast, when trained with the SGD optimizer, the representations {$Z_{2}$, $Z_{3}$} do not show noticeable compression and, likewise, when trained with SGD-momentum optimizers, the representations {$Z_{2}$, $Z_{3}$} also do not exhibit much compression. Note that, in our experiment with the CNN trained on the CIFAR classification task, we can see from Table 4 that the model trained with the RMSprop optimizer achieved the best generalization performance, followed closely by the model trained with Adam. Therefore, it appears that adaptive optimizers—which adjust the learning rate per parameter—may be critical for leading to network compression, and hence generalization [70].

#### 5.3.2. How Does Regularization Impact the Network Dynamics?

It has been suggested by [11,12] that weight decay regularization can significantly enhance the compression phase associated with a neural network learning dynamic. It has also been argued by others [18] that compression is only possible with regularization. Therefore, we also investigated, under the lens of our approach, the effect of regularization on the learning dynamics of MLPs and CNNs.

**Setup:** We deployed MLP 64 × 4 models trained on the MNIST dataset with or without weight decay (WD) regularization and CNN models trained with the CIFAR-10 dataset with or without dropout regularization. The weight decay was applied to all layers in the MLP 64 × 4 model with its hyper-parameter set to 0.001, while the dropout was only adopted in the first fully connected layer in the CNN with a 30%, 60%, or 90% dropout rate (which is a common approach in the literature [54]). The MLP with weight decay regularization requires more epochs to converge. Therefore, we trained the MLP 64 × 4 without weight decay for 300 epochs and the model with weight decay for 1200 epochs.

**Figure 13:**
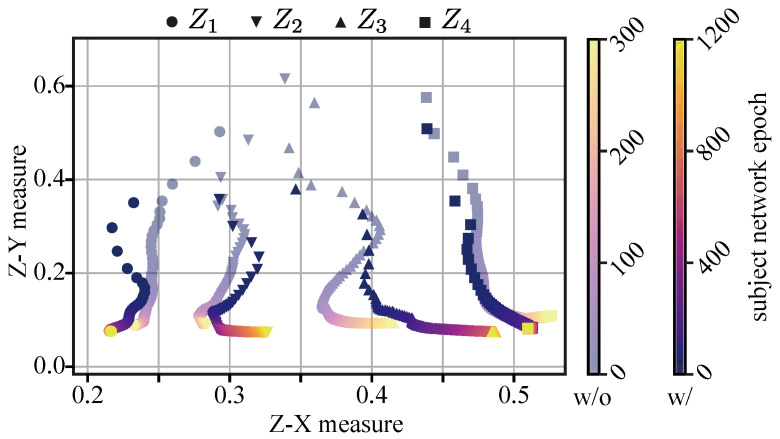
Z-X and Z-Y dynamics of MLP 64 × 4 trained on the MNIST dataset with or without weight decay regularization. The subject network regularized by weight decay gives relatively better test loss. The representations (e.g., $Z_{1}$) are taken from the corresponding block of the MLP 64 × 4 network in Figure 2.

**Results:** We offer the dynamics of the Z-X and Z-Y measures associated with the MLP setting in Figure 13. We infer that weight decay regularization does not significantly impact the fitting phase; however, weight decay does seem to affect network compression, leading networks to compress more aggressively. Moreover, weight decay not only prevents the subject network from overfitting [2] but also prevents its representations from overfitting. Therefore, we conjecture that the weight decay regularization boosts the compression in MLPs (as also observed in [11]) and prevents the representation overfitting to improve the generalization performance (shown in Table 5), which is also in line with [11].

**Figure 14:**
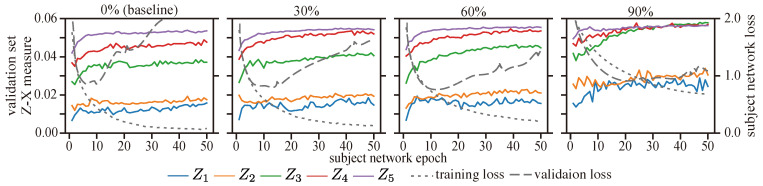
The Z-X dynamics for a CNN trained on the CIFAR-10 dataset with different amounts of dropout in its fully connected layers. The subject network regularized with a 60% dropout rate provides the best test loss and generalization error. The representations (e.g., $Z_{1}$) are taken from the corresponding block of the CNN network in Figure 2.

We also offer the dynamics of the Z-X measure associated with the CNN setting in Figure 14 (Table 5 shows that the best generalization performance is obtained for a CNN with dropout regularization at a 60% dropout rate on the first fully connected layer). Our results suggest that tuning the dropout rate on the first fully connected layer affects not only the dynamics of its representation ($Z_{4}$) but also the dynamics of other layers. When a high dropout rate (e.g., 90%) is used, we observe less pronounced fitting and compression phases, which also lead to a worse generalization performance (refer to Table 5). Conversely, a low dropout rate (30%) showed similar fitting phases to the no-dropout group, but with more compression. These results support our conjecture that the F/C phases are linked to the generalization behavior of the model.

On the other hand, it can be observed that adopting dropout regularization diminishes the visibility of fitting phases across multiple layers. This suggests that the training algorithm effectively leverages the neurons and connections within the model, enabling rapid dataset fitting.

### 5.4. The Impact of Dataset to the Network Dynamics

It is well established that the size of the training set directly affects a machine learning model’s generalization performance [71]. Our goal was to also understand how the dataset size affects neural network model learning dynamics, including its fitting and compression phases.

**Setup:** We compared the learning dynamics of CNN models trained on three different datasets: 1% of CIFAR-10 (0.5k samples), CINIC [56] (which has the same classes as CIFAR-10 but contains 180k samples), and the full CIFAR-10 dataset (50k samples).

We used the Adam optimizer with a learning rate of 0.001 to train the neural networks. We also estimated the Z-X and Z-Y measures using the network in Figure A3 using the CIFAR-10 validation and test sets.

**Figure 15:**
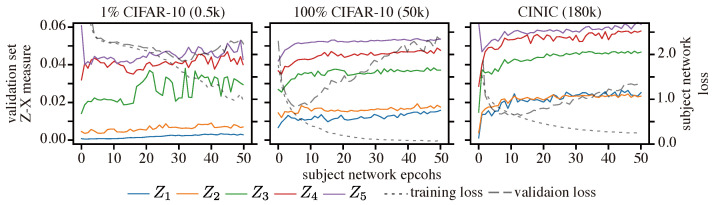
Z-X measures of representations in CNN trained on 1% CIFAR-10 dataset, full CIFAR-10 dataset, and CINIC dataset. The representations (e.g., $Z_{1}$) are taken from the corresponding block of the CNN network in Figure 2.

**Results:** Figure 15 shows the Z-X dynamics of CNNs trained on datasets of different sizes. We can observe from Table 6 that the model trained on the CINIC dataset achieves the best generalization performance, while the model trained on the smallest dataset (1% CIFAR-10) performs the worst.

Our experiments show that the fitting behavior of the network trained on the small dataset is identical to that of the network trained on the standard CIFAR-10 dataset. However, the degree of compression exhibited by the network optimized on the 1% CIFAR-10 dataset was much less pronounced than that of the model trained on richer datasets. This suggests that compression may only be possible for sufficiently large datasets. Our experiments also show that the behavior of the Z-X measure associated with the network trained on the CINIC dataset rapidly increases during the optimization process. This indicates a significant F/C phase that may also justify the superior generalization performance.

Overall, these observations suggest that providing sufficient training data can amplify the magnitude of compression. This in turn helps the model learn to abstract key information for predicting labels more effectively, leading to a better generalization performance. Therefore, we conclude that compression may be a crucial factor for effective generalization in neural networks, and providing sufficient training data is essential for amplifying this phase [8].

## 6. Conclusions

In this paper, we proposed to replace the mutual information measures associated with information bottleneck studies with other measures capable of capturing fitting, compression, and generalization behavior. The proposed method includes: (1) the Z-X measure corresponding to the approximation of the minimum mean-squared error associated with the recovery of the network input (X) from some intermediate network representation (Z) and (2) the Z-Y measure associated with the cross-entropy of the data label/target (Y) given some intermediate data representation (Z). We also proposed to estimate such measures using neural-network-based estimators. The proposed approach can handle representations in high-dimension space, is computationally stable, and is also computationally affordable.

Our series of experiments explored—via the dynamics between the Z-X and Z-Y measure estimates—the interplay between network fitting, compression, and generalization on different neural networks, with varying architectures, learning algorithms, and datasets, that are as complex or more complex than those used in traditional IB studies [12]. Our main findings are as follows:

- **Impact of Neural Network Architecture:**
  − We have found that MLPs appear to compress regardless of the non-linear activation function.
  − We have observed that MLP generalization, fitting, and compression behavior depend on the number of neurons per layer and the number of layers. In general, the MLPS offering the best generalization performance exhibit more pronounced fitting and compression phases.
  − We have also observed that CNN generalization, fitting, and compression behavior also depend on the kernel’s number/size. In general, CNNs exhibiting the best generalization performance also exhibit pronounced fitting and compression phases.
  − Finally, we have seen that the fitting/compression behavior exhibited by networks with residual connections is rather distinct from that shown in networks without such connections.
- **Impact of Neural Network Algorithms:** We have observed that adaptive optimizers seem to lead to more compression/better generalization in relation to non-adaptive ones. Likewise, we have also observed that regulation can help with compression/generalization.
- **Impact of Dataset:** Our main observation is that insufficient training data may prevent a model from compressing and hence generalizing; in turn, models trained with sufficient training data exhibit both a fitting phase followed by a compression phase, resulting in a higher generalization performance.

Overall, our findings are in line with an open conjecture that good neural network generalization is associated with the presence of a neural network fitting phase followed by a compression phase during the learning process [8,11,29].

There are some interesting directions for further research. First, it would be intriguing to explore the dynamics of state-of-the-art machine learning models, including transformers, which have demonstrated exceptional performance in various tasks. By analyzing the behavior of transformers under the lens of the information bottleneck theory, we may be able to gain additional insights into how these advanced models learn, compress information, and generalize.

Second, it would also be interesting to extend the study to other learning paradigms such as semi-supervised or unsupervised tasks. In semi-supervised learning, where a limited amount of labeled data are available along with a larger unlabeled dataset, using the proposed approach to study the learning process may help to uncover effective strategies for leveraging unlabeled data. Similarly, in unsupervised learning tasks, where the goal is to discover patterns and structure in unlabeled data, a similar approach could potentially uncover the interplay between compression and fitting and their implications in leading up to meaningful representations capturing essential information.

Finally, although our study has shed some light on the interplay between compression and generalization using the proposed method, conducting a specialized study and analysis to obtain a more comprehensive understanding of the relationship between these two factors would be interesting.

## Figures and Tables

**Table 1:** Overview of our main results and related literature results. Fit., Com., and Gen. are abbreviations for fitting, compression, and generalization, respectively. Note that the related literature listed explored the information bottleneck under similar setups but may report different observations or focus on different phenomena in the dynamics.

Study	Model	Training Algorithm	Dataset	Section	Our Observation	Related Literature
Effects of model architectures	Tishby-nets with saturated or non-saturated activation functions	SGD	Tishby-dataset	Section 5.2.1	Fit./Com. phases exist regardless of the type of activation function.	[8,11,14,24,26,27]
	MLPs with more or fewer neurons per layer		MNIST	Section 5.2.2	MLPs with more neurons per layer exhibit faster Fit., more Com., and better Gen.	-
	MLPs with more or fewer layers				MLPs with more layers have less Fit. but more Com. The MLP that exhibits more pronounced Fit. and Com. also tends to Gen. better.	[27]
	CNNs with more or fewer kernels	Adam	CIFAR-10	Section 5.2.3	CNNs with fewer kernels cannot Fit. and Com. effectively, and do not Gen. well. Increasing the number of kernels on a well-generalized CNN does not have a significant impact on Fit., Com., or Gen.	-
	CNNs with bigger or smaller kernels				Both very large and very small kernel sizes tend to result in less Fit. and Com., and can harm Gen.	
	ResCNN			Section 5.2.4	The representations at the outputs of residual blocks do not exhibit Fit./Com. phases, while the representations in the residual blocks exhibit Fit./Com. phases.	[13,18,28]
Effects of training algorithms	CNN	SGD, SGD-momentum, RMSprop, Adam		Section 5.3.1	Adaptive optimizers compress more on layers closer to the input.	[29]
	MLP	SGD with or without weight decay	MNIST	Section 5.3.2	Weight decay does not significantly affect the Fit. phase, but it can increase the Com. capability of the model and improve its Gen. performance.	[11,18,21]
	CNN	Adam with or without dropout	CIFAR-10		A low dropout rate does not significantly impact Fit., but it can enhance Com. and improve Gen. In contrast, a high dropout rate can lead to less Fit. and Com., resulting in worse Gen.	-
Effects of dataset size		Adam	CIFAR-10, CINIC	Section 5.4	CINIC dataset enhances Fit., Com., and Gen. CIFAR-10 subset has less Com. and worse Gen.	[8,14]

**Table 2:** The epoch that reached the minimum validation loss (ep.), the training losses, test losses (test loss), generalization error (GE), training accuracy (train acc.), and test accuracy (test acc.) of the MLPs with different widths and depths. The experiment with the best generalization error is highlighted using bold font.

Subject Network	ep.	Train Loss	Test Loss	GE	Train acc.	Test acc.
MLP 16 × 4	197	0.0890	0.1471	0.0581	0.9740	0.9572
MLP 64 × 4	168	0.0344	0.0967	0.0623	0.9919	0.9748
MLP 512 × 4	142	0.0191	0.0697	**0.0506**	0.9967	0.9800
MLP 64 × 2	299	0.0688	0.1247	0.0559	0.9815	0.9760
MLP 64 × 3	275	0.0338	0.0570	**0.0232**	0.9919	0.9762
MLP 64 × 4	142	0.0344	0.0967	0.0623	0.9919	0.9748
MLP 64 × 5	85	0.0659	0.1185	0.0526	0.9822	0.9672
MLP 64 × 6	68	0.0736	0.1320	0.0584	0.9798	0.9616

**Table 3:** The epoch that reached the minimum validation loss (ep.), the training losses, test losses (test loss), generalization error (GE), training accuracy (train acc.), and test accuracy (test acc.) of the CNNs with a different number of kernels and kernel sizes. The experiment with the best generalization error is highlighted using bold font.

Subject Network	ep.	Train Loss	Test Loss	GE	Train acc.	Test acc.
CNN baseline	5	0.6747	0.8300	**0.1553**	0.7657	0.7190
CNN ×2	7	0.3826	0.8303	0.4477	0.8637	0.7514
CNN ×4	4	0.5667	0.7801	0.2135	0.8001	0.7332
CNN /2	11	0.6015	0.9055	0.3040	0.7871	0.7008
CNN /4	14	0.7704	1.0494	0.2790	0.7306	0.6492
CNN /8	26	0.9515	1.1353	0.1838	0.6589	0.6060
CNN 1 × 1	18	1.0307	1.1860	0.1553	0.6343	0.5978
CNN 3 × 3	5	0.6747	0.8065	**0.1318**	0.7657	0.7190
CNN 5 × 5	9	0.6001	0.9805	0.3804	0.7887	0.6958
CNN 7 × 7	6	0.8372	1.2011	0.3639	0.7031	0.6042

**Table 4:** The epoch that reached the minimum validation loss (ep.), the training losses, test losses (test loss), generalization error (GE), training accuracy (train acc.), and test accuracy (test acc.) of the CNNs trained with different optimizers. The experiment with the best generalization error is highlighted using bold font.

Subject Network	ep.	Train Loss	Test Loss	GE	Train acc.	Test acc.
CNN SGD	106	0.0202	0.0429	0.0226	0.9945	0.9882
CNN SGD-momentum	131	0.0130	0.0356	0.0226	0.9972	0.9882
CNN Adam	24	0.0067	0.0275	0.0208	0.9979	0.9896
CNN RMSproop	11	0.0123	0.0263	**0.0139**	0.9965	0.9908

**Table 5:** The epoch that reached the minimum validation loss (ep.), the training losses, test losses (test loss), generalization error (GE), training accuracy (train acc.), and test accuracy (test acc.) of the MLPs and CNNs trained w/ or w/o regularization algorithms. The experiment with the best generalization error is highlighted using bold font.

Subject Network	ep.	Train Loss	Test Loss	GE	Train acc.	Test acc.
MLP w/o WD	168	0.0344	0.0967	0.0623	0.9919	0.9748
MLP w/ WD	626	0.0216	0.0722	**0.0505**	0.9976	0.9784
CNN 0% dropout	5	0.6747	0.8300	0.1553	0.7657	0.7190
CNN 30% dropout	12	0.4993	0.7985	0.2992	0.7608	0.7398
CNN 60% dropout	10	0.6888	0.7606	**0.0718**	0.8237	0.7510
CNN 90% dropout	19	1.0768	0.8765	0.2003	0.5959	0.7000

**Table 6:** The epoch that reached the minimum validation loss (ep.), the training losses, test losses (test loss), generalization error (GE), training accuracy (train acc.), and test accuracy (test acc.) of the CNNs trained on different datasets or dataset sizes, including 1% CIFAR-10 dataset (w/0.5k training samples), 100% CIFAR-10 dataset (w/50k training samples), and CINIC dataset (180k training samples). The experiment with the best generalization error is highlighted using bold font.

Subject Network	ep.	Train Loss	Test Loss	GE	Train acc.	Test acc.
CNN 1% CIFAR-10	38	1.2497	1.9902	0.7405	0.5380	0.3248
CNN 100% CIFAR-10	5	0.6747	0.8300	0.1553	0.7657	0.7190
CNN CINIC	12	0.5952	0.6395	**0.0443**	0.7744	0.7872

**Figure A1:**
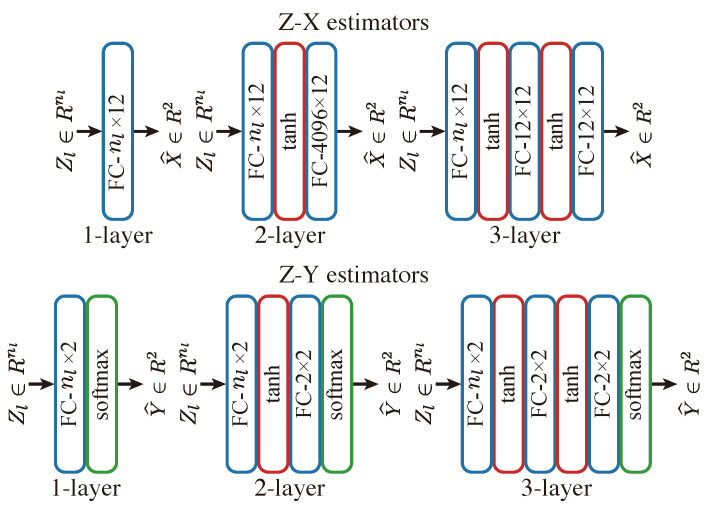
Architecture of the Z-X and Z-Y estimator networks for Tishby-net models. Note that estimators with different depths are used for the stability tests.

**Figure A2:**
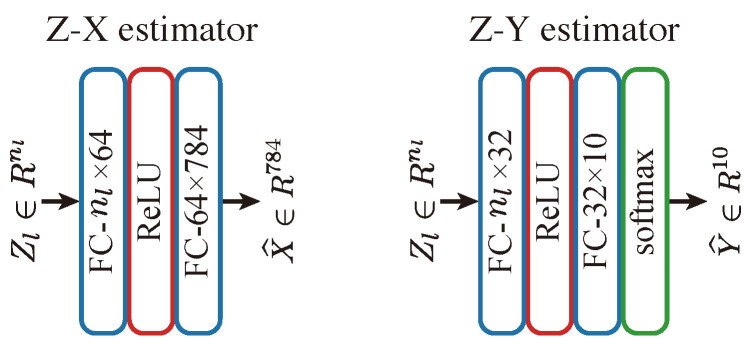
Architecture of the Z-X and Z-Y estimator networks for MLP WxL models.

**Figure A4:**
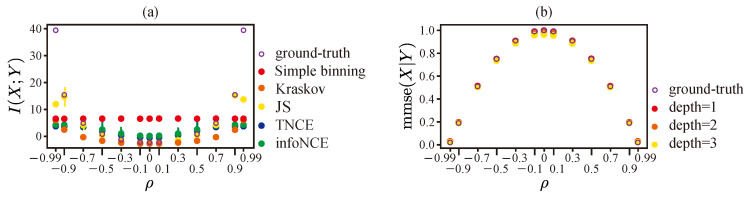
(**a**) The estimated mutual information, and (**b**) the neural network estimated MMSE for 20-dimension correlated Gaussian random variables. In both panels, the hollow purple circles are ground-truth values. In panel (**a**), the infoNCE [10], TNCE [72], and JS [73] are variational estimators, and the error bar is the variance. The simple binning method is adopted via the same implementation as in [24] and the Kraskov estimator is implemented based on the original paper [46]. For panel (**b**), we use a 1-layer (linear), 2-layer, and 3-layer network to estimate the minimal mean-squared error, respectively. The networks have 20 neurons per layer, and the activation function is the hyperbolic tangent function. The estimators are trained with Adam optimizer for 5000 epochs, and the learning rate is set to 0.001.

## Data Availability

Not applicable.

## Appendix A. Estimator Network Architectures

This appendix lists the architectures of the Z-X and Z-Y estimator networks.

## Appendix B. Empirical Comparison of MMSE Estimator and MI Estimator for Multivariant Gaussian Random Variables

We experimentally compared the minimal mean-squared estimator and mutual information estimator for multivariant Gaussian random variables.

Consider a simple case where random vector $X\in R^{d}$ (target) and $Y\in R^{d}$ (observation) follow a multivariate normal distribution with correlation $\Sigma_{XY}=\rho I$, i.e., $Y=\rho X+\sqrt{1-\rho^{2}}N$. Under this setup, the mutual information between *X* and *Y* is $I\left(X;Y\right)=-\frac{d}{2}\log\left(1-\rho^{2}\right)$, and the minimal mean-squared error is $\mathrm{mmse}\left(X|Y\right)=1-\rho^{2}$.

Now, we estimate I(X;Y) with MI estimators from the literature and estimate minimal mean-squared error with neural-network-based mean-squared error estimators. The results are shown in Figure A4. We show the case where d=20 and change ρ from −0.99 to 0.99. Each test takes 4000 randomly generalized samples.

We can see from Figure A4a that the variational estimators tend to have high biases when the mutual information is high. The simple binning method failed to estimate the correct value of mutual information. Although the Kraskov estimator shows a relatively consistent trend, the time consumption of this algorithm grows exponentially as the dimension and number of samples increase [46]. On the other hand, the results of the estimated minimal mean-squared error are shown in Figure A4b. We can see that the estimated values are very close to the ground-truth values.
